# Prolonged rectal shedding of SARS-CoV-2 in a 22-day-old-neonate: a case report

**DOI:** 10.1186/s12887-021-02976-7

**Published:** 2021-11-12

**Authors:** Julie Niemann Holm-Jacobsen, Julia Helena Vonasek, Søren Hagstrøm, Mette Line Donneborg, Suzette Sørensen

**Affiliations:** 1 Centre for Clinical Research, North Denmark Regional Hospital, Bispensgade 37, 9800 Hjoerring, Denmark; 2 Department of Pediatrics, North Denmark Regional Hospital, Hjoerring, Denmark; 3 Department of Pediatrics, Aalborg University Hospital, Aalborg, Denmark; 4 Department of Clinical Medicine, Aalborg University, Aalborg, Denmark

**Keywords:** COVID-19, SARS-CoV-2, Neonate, Rectal shedding, Case report

## Abstract

**Background:**

Severe acute respiratory syndrome coronavirus 2 (SARS-CoV-2) causes the novel coronavirus disease 2019 (COVID-19), which is characterized by a diverse clinical picture. Children are often asymptomatic or experience mild symptoms and have a milder disease course compared to adults. Rectal shedding of SARS-CoV-2 has been observed in both adults and children, suggesting the fecal-oral route as a potential route of transmission. However, only a few studies have investigated this in neonates. We present a neonate with a mild disease course and prolonged rectal SARS-CoV-2 shedding.

**Case presentation:**

A 22-day old neonate was admitted to the hospital with tachycardia and a family history of COVID-19. The boy later tested positive for COVID-19. His heart rate normalized overnight without intervention , but a grade 1/6 heart murmur on the left side of the sternum was found. After excluding signs of heart failure, the boy was discharged in a habitual state after three days of admission. During his admission, he was enrolled in a clinical study examining the rectal shedding of SARS-CoV-2. He was positive for SARS-CoV-2 in his pharyngeal swabs for 11 days after initial diagnosis and remained positive in his rectal swabs for 45 days. Thereby, the boy remained positive in his rectal swabs for 29 days after his first negative pharyngeal swab.

**Conclusions:**

The presented case shows that neonates with a mild disease course can shed SARS-CoV-2 in the intestines for 45 days. In the current case, it was not possible to determine if fecal-oral transfer to the family occurred, and more research is needed to establish the potential risk of the fecal-oral transmission route.

## Background

Severe acute respiratory syndrome coronavirus 2 (SARS-CoV-2) has resulted in a worldwide pandemic since its outbreak in Wuhan, China, in December 2019 [1, 2]. To date, the virus has globally infected more than 230 million people and has led to nearly five million deaths [3]. Infection with SARS-CoV-2 causes the novel coronavirus disease 2019 (COVID-19), which is characterized by a diverse clinical picture. The disease can present with clinical manifestations such as fever, cough, and dyspnea but can also be asymptomatic [4–9]. In critical cases, infection with SARS-CoV-2 may result in respiratory failure, septic shock, multi-organ failure, and/or death [10, 11].

Children most often have a milder disease course compared to adults [9, 10]. They are often asymptomatic or experience mild symptoms, such as fever and cough [9, 12–15], and critical cases are less common [9]. However, a few children, including neonates, develop multisystem inflammatory syndrome (MIS-C) after COVID-19 [16–19].

The primary transmission routes of SARS-CoV-2 are through close contact with infected people and inhalation of respiratory droplets and aerosol particles [20–27]. However, other transmission routes have been suggested, including the fecal-oral route [28–31]. Studies have shown that both adults [28, 32, 33] and children [12, 13, 33–35] can shed SARS-CoV-2 in the stool. It has been estimated that rectal SARS-CoV-2 shedding can be detected, in either stool samples or rectal swabs, in 43 % of COVID-19 patients [36]. Notably, a large fraction of the patients continues to display rectal SARS-CoV-2 shedding, even after the virus has been cleared from the respiratory tract [36]. For children particularly, the prevalence of rectal SARS-CoV-2 shedding is even higher; ranging from 52.6-91.8 % [37–40]. Children have been reported to exert prolonged shedding of SARS-CoV-2 in the intestines for 2-70 days after initial testing with a mean value of 30.8 ± 18.6 days for symptomatic children and 28.1 ± 13.3 days for asymptomatic children [40]. Furthermore, most children continue to be positive in the stool after respiratory samples have become negative [38–40]. Only a few studies have investigated rectal SARS-CoV-2 shedding in neonates [34, 41–43]. Of these, two studies followed the neonates until they became negative in the rectal swabs or stool, which lasted for four and 42 days, respectively [34, 43].

The present case report describes the shedding of SARS-CoV-2 in the pharynx and the rectum of a 22-day-old neonate, to draw further attention to the possible prolonged shedding of SARS-CoV-2 in neonates.

## Case presentation

We present a case of a 22-day old boy who was admitted to the pediatric ward at the North Denmark Regional Hospital in Hjoerring, Denmark, with an active COVID-19 infection.

The boy was born at term, after an uncomplicated pregnancy, with a birth weight of 3470 g, and a normal APGAR score together with an otherwise normal perinatal period. The boy was bottle-fed and well-being with a normal weight gain.

At 21 days of age, the family contacted the pediatric ward because the boy’s father and oldest sister were tested positive for COVID-19, and the boy had been irritable for 2 hours and had a temperature of 38.3 °C. A few hours later, the temperature had dropped to 37.8 °C, and the boy was no longer irritable. Both the father and sister were asymptomatic.

At admission, the boy presented no respiratory difficulties, however, he had tachycardia of 200 beats/minute and was pale. He had a body temperature of 37.3 °C, respiratory rate of 48 breaths/minute, small intercostal retractions, normal stethoscopy of the lungs and heart, and an otherwise normal neonatal examination and vital values. The child underwent a COVID-19 pharyngeal PCR-test, a chest and abdomen x-ray as well as screening blood analyses, and an electrocardiogram (ECG) (Table 1).

**Table 1:** Laboratory findings

	Reference interval	Day 1	Day 2	Day 3
ECG	Sinus rhythm, 100-160 beats/minute	At admission: Sinus rhythm, 190 beats/minute 12 hours after admission: Sinus rhythm, 169 beats/minute		Sinus rhythm, 159 beats/minute
CRP	<8.0 mg/L	<4		<4
Haemoglobin	6.8-12.5 mmol/L	9.6		
Thrombocytes	120-555 10^9^/L	224		262
Leucocytes	6.9-19.9 10^9^/L	10.2		15.3
Creatinine	17-41 μmol/L	21		
Alanine transaminase	1-40 U/L	**57**		
pH^a^	7.37-7.45	7.38		
pCO_2_^a^	3.8 -6.5 kPa	5.7		
Bicarbonate^a^	21.3-26.5 mmol/L	24.3		
Lactate^a^	0.5-1.6 mmol/L	**2.1**		
ProBNP	<300 ng/L	**708**	**785**	
Creatinine kinase	30-470 U/L	139	168	125
Creatinine Kinase MB	<7.0 μg/L	7.0	**8.2**	6.6
Troponin I	No reference values ng/L	48	49	

Numbers in bold indicate values that are outside the reference interval

^a^Capillary samples

The x-ray and ECG were normal. For laboratory findings, see Table 1. Overnight, his heart rate normalized without intervention, and along with this a normalization of skin color was seen. In the morning, the COVID-19 test came back positive. A grade 1/6 heart murmur on the left side of the sternum was found, and a pediatric cardiologist was consulted. It was decided to repeat the ECG and take blood samples for heart enzymes to rule out MIS-C (Table 1). The pediatric cardiologist concluded that there were no signs of heart failure biochemically or clinically. After the first hours, the heart rate was stable at 130-160 beats/minute, although slightly higher when he showed signs of irritability, and he was normothermic. The boy was discharged in a habitual state after three days of admission. A follow-up echocardiography one month after admission was normal. Two days after the boy was discharged, the mother tested positive for COVID-19. To our knowledge, the second sister remained negative.

**Fig. 1:**
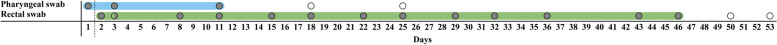
Timeline of results from pharyngeal and rectal swabs analyzed by PCR-based test. Day 1 is the time of COVID-19 diagnosis, and day 2 is the day of inclusion in the clinical study. Grey circles  demonstrate a positive result, transparent circles  demonstrate a negative result, and half-filled circles  demonstrate an inconclusive result. The blue box marks the period of positive pharyngeal swabs, while the green box marks the period of positive rectal swabs

Due to the positive COVID-19 test, the boy was enrolled in a clinical study (N-20200036) at the North Denmark Regional Hospital, examining rectal shedding of SARS-CoV-2. During hospitalization, an extra pharyngeal and two rectal swabs were collected. Prior to discharge, the mother was thoroughly instructed in how to perform pharyngeal and rectal swabs, and was asked to continue sample collection at home, until two consecutive negative pharyngeal and rectal swabs were obtained. Figure 1 shows the number and frequency of samples taken.

Pharyngeal swabs were positive for 11 days after initial diagnosis, and the collection of pharyngeal swabs was stopped after two consecutive negative results on day 25. After this, the mother was instructed to continue with the rectal swabs only. The boy was SARS-CoV-2 positive in his rectal swabs for 45 days, and the sample collection ended on day 53 with two consecutive negative results. Thus, the boy remained positive in his rectal swabs for 29 days after his first negative pharyngeal swab.

At inclusion, the mother completed a questionnaire regarding the boy’s demographic and medical information, including height, weight, symptoms, contact with SARS-CoV-2 infected individuals, risk factors, etc. In addition, the mother completed a questionnaire about the boy’s symptoms at each sample collection time point. After discharge, the boy remained asymptomatic during the whole pharyngeal and/or rectal swab follow-up period. The boy had an average weight gain of 37.6 g/day during the first month after the positive COVID-19 test, hence, the prolonged shedding of virus did not affect the boy’s well-being.

## Discussion and Conclusions

We have presented a case of a 22-day-old COVID-19 neonate with prolonged rectal SARS-CoV-2 shedding. He experienced a mild fever before hospitalization but remained asymptomatic during his involvement in this study. His mild disease course is in line with current reports on clinical manifestations among neonates with COVID-19 [41, 43–46]. The degree and duration of rectal SARS-CoV-2 shedding in neonates is, however, not very well described, and only a few studies have addressed this [34, 41–43]. These studies described neonates with prolonged rectal shedding after initial testing, and most of the neonates had a mild disease course. One of the studies described a neonate with mild symptoms and rectal shedding for 42 days [34]. Therefore, it appears that SARS-CoV-2 resides in the intestines of some neonates, for a relatively long period, without necessarily contributing to the symptoms or severity of the COVID-19 disease. It could be speculated that the combination of mild COVID-19 disease phenotype along with the observed prolongated presence of SARS-CoV-2 in the intestine mirror the unique immune compartment of infancy [47]. However, it is not known if long-term consequences such as influence on gut microbiota establishment could arise or if normal gut function or immune responses could be affected later in life.

The finding of SARS-CoV-2 in stool of neonates could potentially constitute a risk of transferring the virus to close contacts, such as the parents, siblings, or caretakers. Solid evidence for a fecal-oral transmission route for SARS-CoV-2 has not been established, but a few studies have been able to isolate replication-competent virus from stool or rectal swabs [29–31]. In addition, the angiotensin converting enzyme 2 receptor, known to be required for cellular entry of SARS-CoV-2, has been detected on intestinal cells [48, 49]. This, together with the prolonged presence of rectal shedding, supports the likelihood that the virus actively exerts its life cycle in the intestines of COVID-19 patients instead of being non-infectious leftovers. In the current case, it was not possible to determine if any fecal-oral transfer from the neonate to family members occurred since the mother could have been infected during other circumstances.

More research is needed to establish whether prolonged presence of SARS-CoV-2 in neonates could exert a health risk for the child, but also if extra precautionary measures should be taken to prevent viral transmission from stool. The latter, in particular, when considering that a high fraction of COVID-19 positive infants/children present with rectal SARS-CoV-2 shedding and are simultaneously asymptomatic.

## Data Availability

The datasets used and/or analyzed during the current study are available from the corresponding author on reasonable request.

## Abbreviations

COVID-19: Coronavirus disease 2019
ECG: Electrocardiogram
MIS-C: Multisystem inflammatory syndrome in children
SARS-CoV-2: Severe acute respiratory syndrome coronavirus 2
